# Parenchymal lung disease in adult onset Still’s disease: an emergent marker of disease severity—characterisation and predictive factors from *Gruppo Italiano di Ricerca in Reumatologia Clinica e Sperimentale* (GIRRCS) cohort of patients

**DOI:** 10.1186/s13075-020-02245-5

**Published:** 2020-06-22

**Authors:** Piero Ruscitti, Onorina Berardicurti, Daniela Iacono, Ilenia Pantano, Vasiliki Liakouli, Francesco Caso, Giacomo Emmi, Rosa Daniela Grembiale, Francesco Paolo Cantatore, Fabiola Atzeni, Federico Perosa, Raffaele Scarpa, Giuliana Guggino, Francesco Ciccia, Antonio Barile, Paola Cipriani, Roberto Giacomelli

**Affiliations:** 1 Rheumatology Unit, Department of Biotechnological and Applied Clinical Sciences, University of L’Aquila, delta 6 building, PO box 67100, L’Aquila, Italy; 2 Rheumatology Section, Department of Precision Medicine, University of Campania “Luigi Vanvitelli”, Naples, Italy; 3 Rheumatology Unit, Department of Clinical Medicine and Surgery, School of Medicine, University of Naples Federico II, Naples, Italy; 4 Department of Experimental and Clinical Medicine, University of Firenze, Florence, Italy; 5 Department of Health Sciences, University of Catanzaro “Magna Graecia”, Catanzaro, Italy; 6 Rheumatology Unit, Department of Medical and Surgery Sciences, University of Foggia, Foggia, Italy; 7 Rheumatology Unit, Department of Clinical and Experimental Medicine, University of Messina, Messina, Italy; 8 Rheumatic and Systemic Autoimmune Diseases Unit, Department of Biomedical Sciences and Human Oncology (DIMO), University of Bari Medical School, Bari, Italy; 9 Department of Health Promotion, Mother and Child Care, Internal Medicine and Medical Specialties, Rheumatology section, University of Palermo, Palermo, Italy; 10 Diagnostic and Interventional Radiology, Department of Biotechnological and Applied Clinical Sciences, University of L’Aquila, L’Aquila, Italy

**Keywords:** Adult onset Still’s disease, Lung disease, Mortality

## Abstract

**Background:**

Adult-onset Still’s disease (AOSD) is a systemic inflammatory disorder of unknown aetiology usually affecting young adults. Interestingly, recent evidence from the juvenile counterpart of AOSD suggested the emergent high fatality rate of lung disease (LD) in these patients. In this work, we aimed to characterise LD in AOSD, to identify associated clinical features and predictive factors, and to describe long-term outcomes of the disease comparing patients with LD and those without.

**Methods:**

A retrospective assessment of prospectively followed patients, from January 2001 to December 2019, was provided to describe the rate of LD in AOSD, associated clinical features and predictive factors, and long-term outcomes. Patients with AOSD, who were included in *Gruppo Italiano di Ricerca in Reumatologia Clinica e Sperimentale* (GIRRCS) cohort, were assessed.

**Results:**

Out of 147 patients included in GIRRCS cohort, 18 (12.25%) patients were reported to be affected by LD, at the time of diagnosis of AOSD, who were characterised by older age, a higher prevalence of myalgia, of lymph node involvement, of pleuritis, and abdominal pain. Furthermore, patients with LD showed higher values of systemic score and ferritin. Among those clinical variables, older age and systemic score were also independently predictors of LD. Chest CT scans were also obtained, and the most common finding was the peripheral consolidations in 8 (44.4%) patients. Finally, a higher mortality rate, of 38.9%, was registered in patients with LD than others, since it was associated with a significant decreased survival rate.

**Conclusions:**

The presence of LD could suggest an emergent cause of mortality in AOSD, as observed in juvenile counterpart recognising a further marker of severity and poor prognosis to be careful evaluated. Patients with LD were also characterised by some clinical features, higher values of systemic score and ferritin than the others, identifying a subset of patients mostly burdened by systemic signs and symptoms. Although specific designed future studies are needed to fully elucidate the significance of LD in AOSD, a more accurate evaluation and management of this feature could improve the long-term outcomes of these patients.

## Background

Adult-onset Still’s disease (AOSD) is a systemic inflammatory disorder of unknown aetiology usually affecting young adults [1]. AOSD is multigenic autoinflammatory disease, defined at the “crossroads” between autoinflammatory and autoimmune diseases due to the pathogenic involvement of both arms of immune system [2]. Spiking fever, arthritis, and evanescent rash are frequently observed during the disease [3]. Other clinical manifestations include sore throat, elevated liver enzymes, lymphadenopathy, liver involvement, splenomegaly, and serositis [3]. Laboratory tests reflect the systemic inflammatory process showing high levels of erythrocyte sedimentation rate (ESR), C-reactive protein (CRP), and a typical hyperferritinemia [4]. The latter, despite the poor specificity, is considered strongly suggestive of AOSD, also because of its pathogenic role is supposed [5]. Analysing long-term outcomes of AOSD, one third of patients would usually develop a monocyclic pattern, characterised by a systemic single episode, whereas others either a polycyclic pattern, associated with multiple flares alternating with remissions, or a chronic pattern, related to a persistently active disease [6]. Concerning the therapeutic management, it is mainly aimed at targeting pro-inflammatory signs and symptoms of the disease; therefore, glucocorticoids (GCs), synthetic disease-modifying anti-rheumatic drugs (DMARDs), and, in refractory cases, biologic DMARDs, are used to treat these patients [7–9]. However, despite these therapies, patients with AOSD may experience several flares and life-threatening complications, leading to a mortality rate up to 16% [10]. In fact, AOSD is burdened by severe complications, mostly macrophage activation syndrome (MAS), which are characterised by a high mortality rate in adult patients [11]. Interestingly, recent evidence from the juvenile counterpart of AOSD, the systemic-onset juvenile idiopathic arthritis (SJIA), suggested the emergent high fatality rate of lung disease (LD) [12, 13]. Despite this finding, few studies, mainly isolated case reports, analysed LD in AOSD so far [14–16], proposing that nearly 5% of patients with AOSD could be affected by LD, with two main patterns, one characterised by an acute respiratory distress syndrome (ARDS), and one with other LDs, including bronchiolitis and nonspecific interstitial lung diseases [16]. On these bases, given the prognostic role of LD in paediatric patients and the inconclusive evidence in adult ones, we aimed to characterise LD in AOSD and to identify associated clinical features and predictive factors. In addition, by analysis of follow-up, we designed a long-term evaluation to describe outcomes of the disease comparing patients with LD and those without.

## Materials and methods

### Study design, settings, and patients

A retrospective assessment of prospectively followed patients, from January 2001 to December 2019, was provided to describe the rate of LD in AOSD, associated clinical features and predictive factors, and long-term outcomes. Patients with AOSD, who were included in *Gruppo Italiano di Ricerca in Reumatologia Clinica e Sperimentale* (GIRRCS) cohort, were assessed. All patients fulfilled Yamaguchi’s criteria for AOSD [17]. The local Ethics Committee (*Comitato Etico Azienda Sanitaria Locale 1 Avezzano/Sulmona/L’Aquila, L’Aquila*, Italy, protocol number 0139815/16) approved the study, which was performed according to the Good Clinical Practice guidelines and the Declaration of Helsinki.

### Clinical variables and data sources

By analysis of the clinical charts of patients, clinical features, systemic score, occurrence life-threatening complications, therapies, and patterns of disease were reported, as previously detailed [10, 18]. The clinical workup before the AOSD diagnosis considered the exclusion of potential mimickers. We assessed infections by blood cultures, bone marrow cultures (in case of MAS), serology, PCR analyses, chest X-rays, and abdominal echography. We also evaluated the possible differential diagnosis with malignancies using chest X-rays, abdominal echography, and blood samples. Despite these exams, in the case of further suspicion, we added CT and/or PET/CT exams to the diagnostic workup. Thus, chest-X-rays were performed in all patients and in those with further suspicion of pulmonary involvement or the need to exclude infections or malignancies chest CT scans were also performed. LD was defined as AOSD-related parenchymal lung involvement as reported in available literature [12, 13, 16]. In patients with suspicion of LD, chest CT scan was performed, and findings codified according to available literature [12, 13] in different main patterns: (i) multilobar, predominantly peripheral septal thickening, parahilar, and/or anterior upper lobes with or without adjacent ground glass opacities; (ii) crazy-paving; (iii) peripheral consolidations; (iv) peribronchovascular consolidations; (v) predominantly ground-glass opacities [12, 13]. Crazy paving was referred to the appearance of ground-glass opacity with superimposed interlobular septal thickening and intralobular septal thickening, seen on chest CT. Before diagnosing LD related to AOSD, all other possible mimickers were excluded, with more attention to the infectious diseases, as previously performed in SIJA and AOSD [10, 12, 13, 18]. Furthermore, the occurrence of MAS and other AOSD life-threatening complications were assessed, as previously reported [19–21]. Patients in remission were defined as those achieving a complete disappearance of any clinical and laboratory feature of the disease. Inflammatory markers, including ESR, CRP, and ferritin were recorded. At the end of follow-up, patients were categorised into three different disease courses, monocyclic, polycyclic, and chronic patterns, as previously performed [10]. The administration of therapies, glucocorticoids (GCs), synthetic, and biologic DMARDs, for managing AOSD was also registered and categorised, at the time of diagnosis and during the follow-up, as already done [22, 23].

### Statistical methods

Statistics firstly provided the descriptive analysis, and collected results were presented as mean and standard deviation (SD) or median and interquartile range (IQR) according to their distribution. Clinical characteristics of patients with and without LD were compared by parametric or non-parametric *t* tests for all the continuous variables, and chi-squared test was used for the categorical ones, as appropriate. Possible correlations among the presence of LD and ESR, CRP, ferritin, and systemic score were estimated by using a point-biserial coefficient correlation. Furthermore, regression analyses were performed to assess possible predictive factors of LD presence. Kaplan-Meier curves were plotted to determine the rates of survival of patients with AOSD with and without LD; the difference between curves was determined by the log-rank (Mantel-Cox) test. Additionally, Cox regression analysis for survival was performed to evaluate the role of LD on mortality of those patients. Due to the relatively simple study design, few retrieved missing data were managed by exclusion of these from analyses. Two-sided *P* values < 0.05 were considered as being statistically significant. The Statistics Package for Social Sciences (SPSS for Windows, version 17.0, SPSS Inc., Chicago, IL, USA) was used for all analyses.

## Results

### Clinical characteristics of patients with LD

**Fig. 1:**
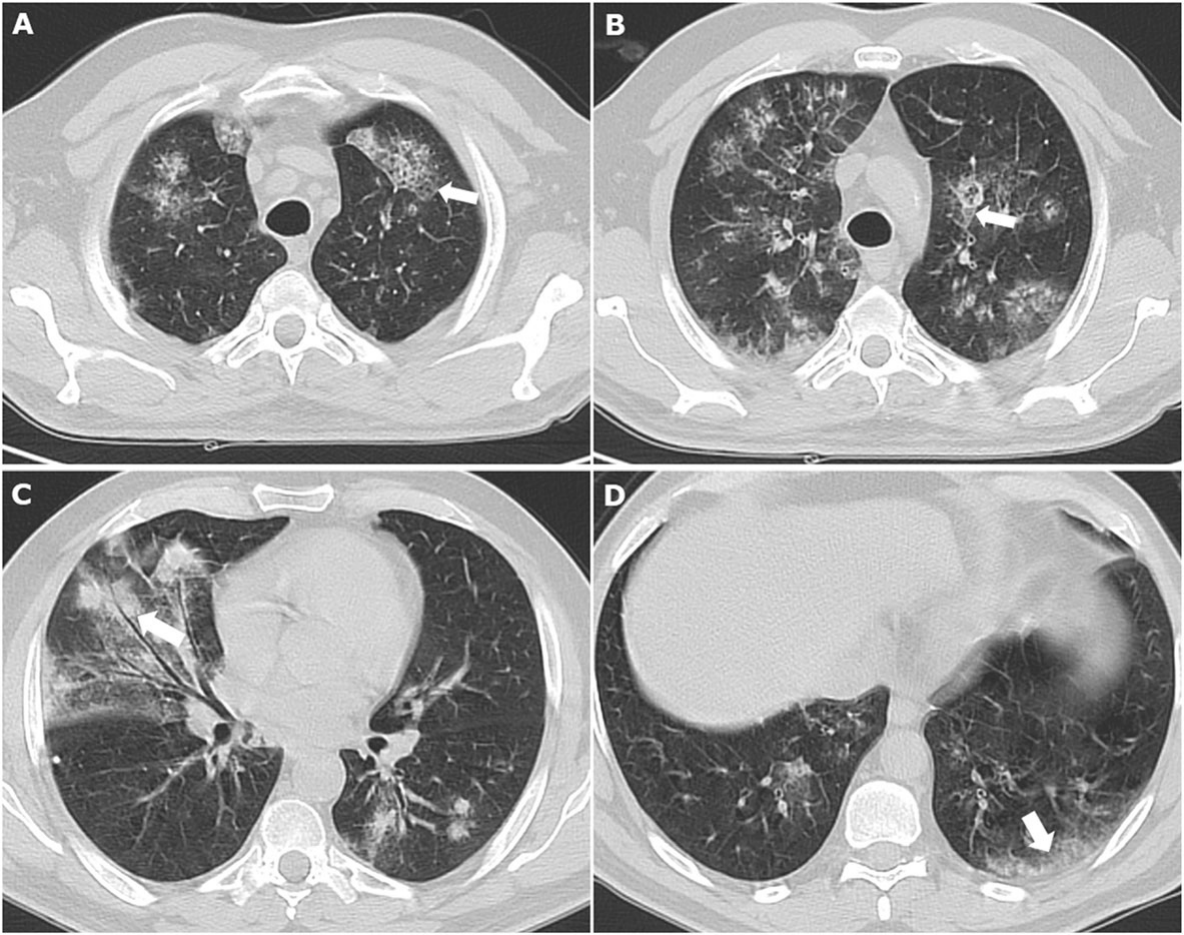
In this figure, different radiological features of LD, present at the same time, in a patient with AOSD are shown as follows: **a** crazy paving (white arrow), **b** crazy paving (white arrow), **c** peribronchovascular consolidations with ground glass, and **d** peripheral consolidation

**Fig. 2:**
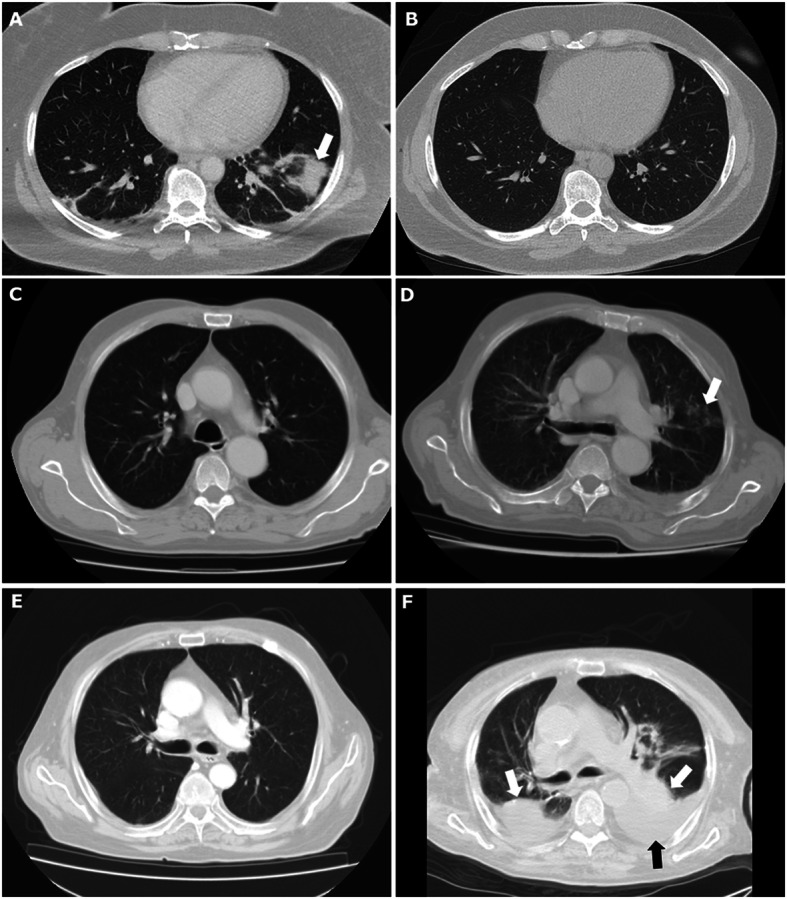
In this figure, radiological features of LD, in patients with AOSD, before and after therapies are shown. In panel **a**, a peribronchovascular consolidation is shown (white arrow) which is completely disappeared after therapy with high dosage of GCs, as shown in panel **b**. In panel **d**, the occurrence of ground glass opacities is reported, after previous negative findings, as shown in panel **c**. In panel **f**, the occurrence of peripheral consolidations (white arrows) with concomitant pleural effusion (black arrow) is reported, after previous negative findings, as shown in panel **e**

Out of 147 patients included in GIRRCS cohort, 18 (12.25%) patients were reported to be affected by LD, at the time of diagnosis of AOSD. Out of these 18 patients, 8 (44.44%) reported smoking habit, presently or formerly. As detailed in Table 1, these patients were characterised by older age (*p* = 0.017), a higher prevalence of myalgia (*p* = 0.033), of lymph node involvement (*p* = 0.028), of pleuritis (*p* <  0.001), and abdominal pain (*p* = 0.001). Furthermore, patients with LD showed higher values of systemic score (*p* <  0.001) and ferritin (*p* = 0.010). In fact, systemic score (coefficient 0.347, *p* <  0.001) and ferritin (coefficient 0.148, *p* = 0.031) were significantly correlated with LD, whereas ESR (coefficient 0.014, *p* = 0.843) and CRP (coefficient 0.084, *p* = 0.220) did not. In patients with LD, respiratory signs and symptoms were mostly subtle, including mild tachypnoea, dyspnoea, and chronic cough, with a discrepancy between clinical picture and extension of lung involvement. The most common CT finding was the peripheral consolidations in 8 (44.4%) patients, less frequently peribronchovascular consolidations in 4 patients (22.2%), predominantly ground glass opacities in 3 patients (16.7%), crazy-paving in 3 patients (16.7%), and multilobar predominantly peripheral septal thickening in 2 patients (11.1%). Most showed one or more of these patterns, which appeared to be not mutually exclusive. In almost all patients, 14 out of 18 (77.8%), a follow-up evaluation of chest CT scan was retrieved, but analysed without any inferential analysis due to the lack of power. In patients, who improved, a complete disappearance of inflammatory findings was reported, whereas in others a worsening of LD was observed. In Fig. 1, representative images of chest CT findings of LD related AOSD are shown, whereas in Fig. 2, disappearance or worsening of inflammatory signs are reported after therapy. In these patients, no evidence of clinical pulmonary hypertension, of ARDS, and clubbing was reported. Finally, unusual adverse drug reactions were not retrieved.

**Table 1:** Descriptive statistics of clinical characteristics of assessed patients with AOSD and grouped according to the presence of LD

*Clinical characteristics*	*18 patients with LD*	*129 patients without LD*	*P values, patients with LD* vs *patients without LD*
Age, mean ± SD	53.6 ± 14.7 years	44.0 ± 16.1 years	**0.017**
Gender, *n* (%)	9 (50.0) male	80 (62.0) male	0.441
Fever, *n* (%)	18 (100.0)	129 (100.0)	/
Arthritis, *n* (%)	16 (88.9)	114 (88.4)	0.654
Skin Rash, *n* (%)	13 (72.2)	97 (75.2)	0.492
Myalgia, *n* (%)	16 (88.9)	79 (61.2)	**0.033**
Splenomegaly, *n* (%)	14 (77.8)	84 (65.1)	0.215
Lymph node involvement, *n* (%)	14 (77.8)	66 (51.2)	**0.028**
Sore throat, *n* (%)	12 (66.7)	71 (55.0)	0.450
Liver Involvement, *n* (%)	11 (61.1)	80 (62.0)	0.567
Pleuritis, *n* (%)	10 (55.5)	19 (14.7)	**< 0.001**
Pericarditis, *n* (%)	7 (38.9)	24 (18.6)	0.064
Abdominal Pain, *n* (%)	8 (44.4)	12 (9.3)	**0.001**
Systemic score, mean ± SD	8.4 ± 2.1	5.7 ± 1.8	**< 0.001**
MAS, *n* (%)	5 (27.8)	21 (16.3)	0.071
Leucocytosis > 15,000/mm^3^, *n* (%)	11 (61.1)	67 (51.9)	0.318
ESR, mean ± SD	70.7 ± 24.7 mm/hr	69.2 ± 27.7 mm/hr	0.821
CRP, median (interquartile range)	113.1 (99.2) mg/L	77.0 (79.6) mg/L	0.080
Ferritin, median (interquartile range)	3370.5 (3051.8) ng/mL	2841.5 (3545.1) ng/mL	**0.010**
Low dosage GCs (≤ 0.5 mg/kg/day), *n* (%)	4 (22.2)	57 (44.2)	0.062
High dosage GCs (> 0.5 mg/kg/day), *n* (%)	14 (77.8)	72 (55.8)	0.789
Synthetic DMARDs, *n* (%)	11 (61.1)	84 (65.1)	0.464
Biologic DMARDs, *n* (%)	5 (27.8)	39 (30.2)	0.536
Monocyclic disease pattern, *n* (%)	4 (22.2)	47 (36.4)	0.510
Polycyclic disease pattern, *n* (%)	3 (16.7)	46 (35.6)	**0.044**
Chronic disease pattern, *n* (%)	4 (22.2)	34 (26.3)	0.479
Mortality, *n* (%)	7 (38.9)	13 (10.1)	**0.004**
Time of follow-up, median (interquartile range)	2.8 (3.4) years	3.9 (3.4) years	0.199

*MRI* magnetic resonance imaging, *AOSD* adult onset Still’s disease, *n* number of patients, *DMARDs* disease-modifying anti-rheumatic drugs, *MAS* macrophage activation syndrome, *ESR* erythrocyte sedimentation rate, *CRP* C-reactive protein, *GCs* glucocorticoids. Bolded values are statistically significant (*p* < 0.05)

### Predictive factors of LD

Considering the prognostic role of LD presence, at the time of diagnosis, possible predictive factors of this feature were tested. By univariate analyses, age (OR = 1.04, 95%CI = 1.01–1.07, *p* = 0.021), myalgia (OR = 5.06, 95%CI = 1.12–22.97, *p* = 0.036), lymph node involvement (OR = 3.34, 95%CI = 1.04–10.69, *p* = 0.042), pleuritis (OR = 7.34, 95%CI = 2.53–20.67, *p* <  0.001), abdominal pain (OR = 7.80, 95%CI = 2.59–23.51, *p* <  0.001), and systemic score (OR = 2.14, 95%CI = 1.54–2.97, *p* <  0.001) were retrieved to be significantly predictive on the likelihood of LD presence. Based on the results of univariate analyses, a multivariate regression model was built to evaluate the independent predictive role of selected variables (age and systemic score) on the likelihood of LD presence, at the time of diagnosis (Table 2). Despite the significant results of univariate analyses, we selected the systemic score to be added in the multivariate analysis since including all other clinical variables and due to the low number of patients with LD. The multivariate analysis showed that older age (OR = 1.04, 95%CI 1.01–1.08, *p* = 0.044) and systemic score (OR = 2.08, 95%CI = 1.50–2.88, *p* <  0.001) were independently predictors on the likelihood of LD presence. Considering the reported prognostic role of systemic score ≥ 7 on mortality in AOSD [10], we tested systemic score also as dichotomy (“yes systemic score ≥ 7”/“no systemic score ≥ 7). Systemic score ≥ 7 resulted to be a significant predictor on the likelihood of LD presence, in univariate analysis (OR = 19.16, 95%CI = 4.20–87.41, *p* < 0.001) as well as in multivariate analysis (OR = 23.31, 95%CI = 4.85–42.08, *p* < 0.001).

**Table 2:** Regression analyses of predictive factors on the likelihood of LD presence in AOSD

Clinical variables	OR	95%CI	*p*
*Univariate analyses*			
Age	1.04	1.01–1.07	**0.021**
Male gender	0.61	0.23–1.65	0.332
Arthritis	1.05	0.22–5.04	0.95
Skin Rash	0.86	0.28–2.59	0.786
Myalgia	5.06	1.12–22.97	**0.036**
Splenomegaly	1.87	0.58–6.03	0.290
Lymph node involvement	3.34	1.04–10.69	**0.042**
Sore throat	1.63	0.58–4.62	0.355
Liver Involvement	0.96	0.35–2.65	0.941
Pleuritis	7.34	2.53–20.67	**< 0.001**
Pericarditis	2.78	0.98–7.92	0.060
Abdominal pain	7.80	2.59–23.51	**< 0.001**
Systemic score	2.14	1.54–2.97	**< 0.001**
Systemic score ≥ 7	19.16	4.20–87.41	**< 0.001**
MAS	1.39	0.42–4.62	0.592
Leucocytosis > 15,000/mm^3^	1.45	0.53–3.99	0.467
ESR	1.01	0.98–1.02	0.819
CRP	1.32	0.85–2.06	0.214
Ferritin	1.48	0.95–2.31	0.083
Low dosage of GCs	0.36	0.11–1.16	0.086
Synthetic DMARDs	0.84	0.30–2.32	0.739
Biologic DMARDs	0.88	0.29–2.66	0.831
Monocyclic Pattern	0.87	0.31–2.48	0.798
Polycyclic disease pattern	0.23	0.05–1.02	0.064
Chronic disease pattern	0.80	0.25–2.59	0.708
*Multivariate analyses*			
Age	1.04	1.01–1.08	**0.044**
Systemic score	2.08	1.50–2.88	**< 0.001**
*X*^2^ = 28.72, *p* < **0.001**			
Age	1.05	1.01–1.09	**0.011**
Systemic score ≥ 7	23.31	4.85–42.08	**< 0.001**
*X*^2^ = 23.06, *p* < **0.001**			

*MRI* magnetic resonance imaging, *AOSD* adult onset Still’s disease, *ESR* erythrocyte sedimentation rate, *CRP* C-reactive protein, *GCs* glucocorticoids, *DMARDs* disease-modifying anti-rheumatic drugs. Bolded values are statistically significant (*p* < 0.05)

### Survival rate of patients with LD

**Fig. 3:**
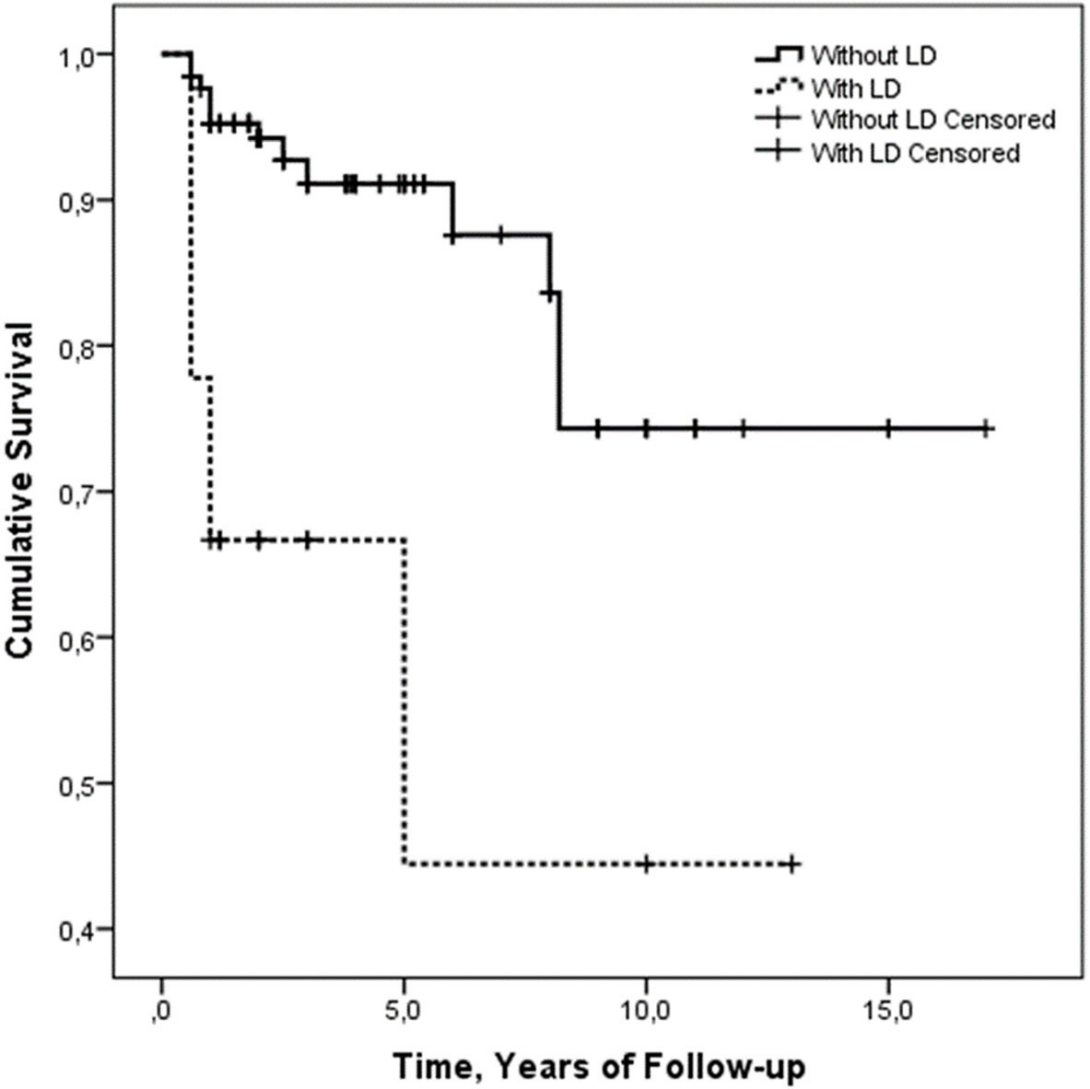
Kaplan-Meier curves of survival in patients with or without LD. The presence of LD was significantly associated with a decreased survival rate of patients with AOSD (*p* < 0.001)

A higher mortality rate (*p* = 0.004), of 38.9%, was registered in patients with and LD than the others. In these patients, no specific chest CT scan pattern was retrieved. Specifically, 5 patients died from uncontrollable MAS, 1 from multiple organ failure with the liver and kidney failures, and 1 from severe infection related to the immunosuppressive therapy, whereas no death due to ARDS was registered in our cohort. The presence of LD, at the time of diagnosis, was significantly associated with a decreased survival rate of patients (*p* < 0.001) during the follow-up, as shown in Fig. 3. Furthermore, the presence of LD, at the time of diagnosis, significantly predicted the mortality in both univariate (HR = 4.81, 95%CI = 1.91–12.12, *p* = 0.001) and in age- and gender-adjusted multivariate (HR = 3.41, 95%CI = 1.28–9.12, *p* = 0.014) analyses for survival.

## Discussion

In the present evaluation, the assessment of LD was performed in a large cohort of patients with AOSD, analysing associated clinical characteristics, predictive factors, and long-term outcomes. Patients with LD were characterised by some clinical features, higher values of systemic score and ferritin, than those without. Some clinical variables, older age and systemic score, were retrieved to be clinical independent predictors on the likelihood of LD presence. Analysing the follow-up, a higher mortality rate was observed in patients with LD, thus identifying an additional marker of severity and poor prognosis to be careful evaluated in AOSD.

In this cohort, 12.25% of patients with AOSD were reported to be affected by LD. This result could suggest a higher rate than reported in available literature [16], but it must be pointed out that mainly isolated case reports investigated this issue [14–16], thus limiting the validity of this estimation in adults. Clinically, as observed in SJIA, patients with AOSD-related LD showed a striking dissociation between relatively subtle clinical features, including mild tachypnoea, dyspnoea, chronic cough, and the extension of the inflammatory process in lungs based on chest CT. Furthermore, patients with LD were characterised by a higher prevalence of myalgia, of lymph node involvement, of pleuritis, and abdominal pain, than others. In addition, those patients showed higher values of systemic score and ferritin. Except from the higher levels of ferritin, different clinical characteristics were retrieved comparing patients with LD with either SIJA or AOSD [12, 13]. In fact, we did not observe any evidence of clinical pulmonary hypertension, of ARDS, clubbing, and unusual adverse drug reactions. In spite of a continuum of the disease is proposed between paediatric and adult patients [24], some dissimilarities, from both clinical and pathogenic point of views, are reported [25–27], suggesting the need of further studies, specifically designed and adequately powered, to fully investigate this issue. In the present evaluation, findings of chest CT scan were analysed and the most common pattern was the peripheral consolidations in 44.4% of patients, differently from what observed in paediatric patients [12, 13].

Furthermore, despite the lack of power, the results about chest CT scans, which were obtained after the therapies, suggested that LD could also follow the activity of the disease, reducing or enhancing according to the achievement of remission or not. Analysing predictive factors of LD, at the time of diagnosis, older age, and higher systemic score resulted to be independent predictors of LD in AOSD. The predictive role of older age seems to be conflicting with findings on paediatric ages, possibly suggesting the role of environmental or occupational factors, such as smoking history or specific dietary habit, to be studied in worsening the course of the disease. Systemic score is a prognostic clinical tool which may be applied to patients with AOSD, identifying, at the time of diagnosis, those patients with the likelihood of a more severe outcome [10]. In addition, a cut-off at 7.0 of the systemic score would identify those patients at higher risk of AOSD-related death, confirming that the multi-organ involvement at the time of diagnosis is a predictive factor of a more severe outcome and increased mortality [10, 25]. Taking together all these features about LD in AOSD, a subset of patients could be identified, mostly characterised by a systemic signs and symptoms of the disease.

Analysing the follow-up of these patients, the presence of LD reduced the survival rate of patients with AOSD, since associated with about 40% of mortality, mirroring what observed in SJIA [12, 13, 28, 29], and suggesting a careful evaluation of this feature. In these patients, no specific chest CT scan pattern was retrieved, suggesting that further studies are needed to fully elucidate this issue. The higher mortality rate observed in our study did not confirm results from other European AOSD series [30, 31], but paralleled with data reported on Asiatic populations [32, 33]. In patients with LD, the main cause of death was the occurrence of uncontrollable MAS and not the occurrence of ARDS. This result mirrored the association between LD and MAS reported in SJIA [12, 13]. In those patients, it has been suggested that the lung involvement may trigger the systemic inflammation and the development of this hyperinflammatory complication [34, 35]. In this context, the IFN-γ may play a central pathogenic role. In fact, in biopsies of lung in patients with SIJA, the analysis of expressed genes revealed that many of the upregulated targets were in gene pathways related to the IFN-γ response, including HLA–D family members and other IFN-related genes [13]. Furthermore, two of the most highly upregulated non-HLA genes were CXCL9 and CXCL10 [13], IFN-induced chemokines strongly correlated with the occurrence of MAS [36, 37]. In addition, the lung is one of the major physiological producers of IL-1 and IL-6, which are also involved in pathogenic steps leading to occurrence of MAS [34, 35, 38, 39]. Thus, it is possible to suggest that the lung involvement observed in these patients, either SIJA or AOSD, could act as a trigger to excessively amplify the immune response, leading to a massive release of pro-inflammatory mediators and, finally, to the occurrence of MAS.

Despite providing an analysis of LD in AOSD, the present evaluation is affected by different limitations associated with the retrospective design, which is affected by different biases. In addition, due to the retrospective design, we did not have contrast enhancement CT scan and lung biopsies, as in paediatric patients [12, 13]. Furthermore, considering the retrospective design and the discrepancy between subtle symptoms and the extension of LD, it is also possible that the first line assessment with chest X-rays could have miss a percentage of patients by minimal pulmonary symptoms. However, although our results should be carefully generalised, it must be pointed out that the rarity of AOSD would make difficult to arrange prospective studies. This is a common unmet need when dealing with rare diseases [40, 41], thus suggesting the significance of retrospective studies to identify relevant clinical features in generating hypotheses to be subsequently investigated and confirmed.

## Conclusions

In conclusion, the presence of LD could suggest an emergent cause of mortality in AOSD, as observed in SJIA, recognising a further marker of severity and poor prognosis to be careful evaluated. Patients with LD were also characterised by some clinical features, higher values of systemic score and ferritin than the others, identifying a subset of patients mostly burdened by systemic signs and symptoms. Although specific designed future studies are needed to fully elucidate the significance of LD in AOSD, a more accurate evaluation and management of this feature could improve the long-term outcomes of these patients.

## Data Availability

All data relevant to the study are included in the article.

## Abbreviations

AOSD: Adult-onset Still’s disease
ESR: Erythrocyte sedimentation rate
CRP: C-reactive protein
GCs: Glucocorticoids
DMARDs: Disease-modifying anti rheumatic drugs
MAS: Macrophage activation syndrome
SJIA: Systemic-onset juvenile idiopathic arthritis
LD: Lung disease
ARDS: Acute respiratory distress syndrome
GIRRCS: Gruppo Italiano di Ricerca in Reumatologia Clinica e Sperimentale
SD: Standard deviation
IQR: Interquartile range
